# A cross-country comparison of health-related quality of life in the United States, Sweden, and Norway during the first year of the COVID-19 pandemic

**DOI:** 10.1186/s13690-023-01088-1

**Published:** 2023-04-20

**Authors:** Jiahe Chen, Cynthia L. Gong, Ulf Persson, Ning Yan Gu

**Affiliations:** 1 Schaeffer Center for Health Policy & Economics, University of Southern California, Los Angeles, CA USA; 2 Department of Pharmaceutical & Health Economics, School of Pharmacy, University of Southern California, Los Angeles, CA USA; 3 Fetal & Neonatal Institute, Children’s Hospital Los Angeles, Los Angeles, CA USA; 4 Department of Pediatrics, Keck School of Medicine, University of Southern California, Los Angeles, CA USA; 5 The Swedish Institute for Health Economics, Lund, Sweden; 6 School of Nursing and Health Professions, University of San Francisco, Sacramento, CA USA

**Keywords:** COVID-19, Health-related quality of life, EQ-5D-5L, Mitigation policy, Cross-country comparison

## Abstract

**Background:**

Limited studies have directly compared health-related quality of life (HRQoL) in different countries during the COVID-19 global pandemic. The objective of this study was to evaluate the HRQoL outcomes in the US, Sweden, and Norway during the first year under the pandemic.

**Methods:**

In April 2020, during early phase of the pandemic, separately in the US, Sweden, and Norway, we surveyed 2,734, 1,003 and 1,020 respondents, then again in January 2021, we collected 2,252, 1,013 and 1,011 respondents. The survey was first developed in English and translated into Swedish and Norwegian. Selected variables were used for the current study. We collected respondents’ HRQoL using the EQ-5D-5L. Respondents’ background information included their sociodemographic data, medical history, and COVID-19 status. We reported the EQ-5D-5L utility, EQ-VAS, and the proportion of problems with each of the EQ-5D-5L health subdomains. Population quality-adjusted life year (QALY) changes based on EQ-5D-5L utility scores were also calculated. Outcomes were stratified by age. One-way ANOVA test was used to detect significant differences between countries and Student’s t-tests were used to assess the differences between waves.

**Results:**

Respectively for the US, Sweden, and Norway, mean EQ-5D-5L utilities were 0.822, 0.768, and 0.808 in April 2020 (*p* < 0.001); 0.823, 0.783, and 0.777 in January 2021 (*p* < 0.001); mean EQ-VAS scores were 0.746, 0.687, and 0.692 in April 2020 (*p* < 0.001), 0.764, 0.682, and 0.678 in January 2021 (*p* < 0.001). For both waves, EQ-5D-5L utilities and EQ-VAS scores in the US remained higher than both Sweden and Norway (*p* < 0.001). Norwegians reported considerably lowered HRQoL over time (*p* < 0.01). Self-reported problems with anxiety/depression were highest for the US and Sweden, while Norwegians reported most problems with pain/discomfort, followed by anxiety/depression. The population QALYs increased in the US and Sweden, but decreased in Norway.

**Conclusions:**

In the first year of the pandemic, a rebound in HRQoL was observed in the US, but not in Sweden or Norway. Mental health issues during the pandemic warrant a major public health concern across all 3 countries.

## Introduction

The COVID-19 pandemic has resulted in significant social, economic, and health consequences throughout the world. Since the beginning of the global pandemic, many studies have evaluated clinical and economic outcomes of COVID-19, with a continued growing body of literature evaluating health-related quality of life (HRQoL) during the pandemic [1–8]. While the impact of the pandemic is global, country-specific differences cannot be ignored [5, 7]. To date, limited studies have performed direct country comparisons using prospectively collected survey data.

The US, Sweden, and Norway each imposed different mitigation strategies and public health policies to reduce COVID-19 transmission. During the early stage of the pandemic on April 1^st^, 2020, for instance, Norway instituted the strictest lockdown and social distancing policies among the three countries, followed by the US, while Sweden implemented the least strict policies [9]. In the US, differing policies were implemented at different times by different states with county-specific regulations resulting in significant regional variation [9–12]. At the same time, Norwegian policies were uniformly imposed by national authorities, regardless of regional or local policies. On the other hand, Swedish regional or local authorities had more discretion, compared with Norwegians, to decide and implement different policies, although still under the guidance of the central public health agency [9, 13].

Because of differing mitigation strategies, we sought to assess and compare the country-specific differences in population HRQoL in these countries and to explore how different lockdown and social distancing policies might be correlated with population HRQoL outcomes in these countries during the first year of the pandemic.

## Methods

### Data

We collected respondents’ sociodemographic data, medical history, COVID-19 status, changes in employment, spending behavior, household incomes and HRQoL as measured by the EQ-5D-5L. US data were collected using the Amazon MTurk, a crowdsourced online platform hosted by Amazon [3]. US adults can register on Amazon Mturk as “workers” to voluntarily complete the questionnaire during the study period. To reimburse the time costs, “Workers” who participated in our survey were compensated $1.50 for each completed survey. The age and gender of the respondent population were controlled to be similar to those of US general population [3]. The same survey was translated into Swedish and Norwegian by native speakers with minor country-specific adaptions. Data collection in Sweden and Norway was conducted by a Swedish survey company, Enkätfabriken, which routinely collects data from nationally representative samples [14]. The survey was sent by Enkätfabriken to samples randomized and stratified based on gender, age and place of residence to be representative of the adult general population in Sweden and Norway. Different samples were recruited for each wave, and a respondent would not receive more than one questionnaire throughout the survey [14]. In each wave, the data were collected in all 3 countries until around 1,000 responses were recorded in Sweden and Norway. The current study used data collected from April 1^st^ to May 6^th^, 2020 (wave 1), and from January 10^th^ to March 15^th^, 2021 (wave 2)(Table 1).

**Table 1:** Selected characteristics of survey participants stratified by country and survey wave

	**N**	**Mean Age (SD)**	**Mean EQ-5D-5L Utilities (SD)**	**Scaled Mean EQ-VAS (SD)**	**Fear on Health (SD)**	**Fear on Financial Situation (SD)**
**United States**						
**Apr 1**^**st**^** – May 6**^**th**^**, 2020 (Wave 1)**	2,734	42.6 (14.3)	0.822 (0.222)	0.746 (0.192)	5.20 (2.95)	5.79 (3.00)
**Jan 10**^**th**^** – Mar 15**^**th**^**, 2021 (Wave 2)**	2,252	42.6 (13.9)	0.823 (0.221)	0.764 (0.186)	5.50 (3.07)	5.36 (3.13)
***p-value (t-test)***		0.9447	0.7904	< 0.001	< 0.001	< 0.001
**Sweden**						
**Apr 1**^**st**^** – May 6**^**th**^**, 2020 (Wave 1)**	1,003	47.8 (17.1)	0.768 (0.260)	0.687 (0.214)	4.65 (2.88)	4.49 (2.89)
**Jan 10**^**th**^** – Mar 15**^**th**^**, 2021 (Wave 2)**	1,013	46.7 (16.6)	0.783 (0.237)	0.682 (0.203)	4.36 (2.64)	3.86 (2.75)
***p-value (t-test)***		0.1541	0.1785	0.5638	0.0206	< 0.001
**Norway**						
**Apr 1**^**st**^** – May 6**^**th**^**, 2020 (Wave 1)**	1,020	46.9 (17.0)	0.808 (0.248)	0.692 (0.208)	4.05 (2.77)	3.82 (2.74)
**Jan 10**^**th**^** – Mar 15**^**th**^**, 2021 (Wave 2)**	1,011	46.9 (16.6)	0.777 (0.271)	0.678 (0.217)	3.88 (2.65)	3.29 (2.69)
***p-value (t-test)***		0.9673	0.0067	0.1241	0.1656	< 0.001
***p-value (ANOVA)***			< 0.001	< 0.001	< 0.001	< 0.001

*p*-value (t-test) was comparing wave 1 and wave 2 outcomes within each country

*p*-value (ANOVA) test was to compare the difference between the 3 countries for each wave

### The EQ-5D-5L

The EQ-5D-5L was used to assess HRQoL measures and changes over time. The EQ-5D-5L is a highly utilized and globally validated HRQoL measure that can be used for online data collection [15]. Respondents rate five health subdomains of the EQ-5D-5L on mobility, self-care, usual activities, pain/discomfort, and anxiety/depression using a Likert scale from 1 (no problems) to 5 (extreme problems). The EQ-5D-5L subdomain responses were used to calculate EQ-5D-5L index scores using existing country-specific algorithms to enable comparability between observed health state utility values that range from 0 (death) to 1 (full health) [16, 17]. The Visual Analog Scale (EQ-VAS), as part of the EQ-5D-5L descriptive system, is also used to assess respondents’ overall health today using a scale of 0–100 (0 = worst imaginable health, 100 = best imaginable health) [15]. In this study, we rescaled the EQ-VAS scores from 0–100 to 0–1 for ease of comparison with utility scores. We then compared the findings across the three countries and between the two waves to determine whether there were any significant differences in HRQoL and other outcomes.

### Analysis plan

Data from the 3 countries and 2 waves were compared in a pairwise fashion. One-way analysis of variance (ANOVA) was used to detect significant differences between countries. Two-way t-tests were used for differences between waves. Results with a *p*-value < 0.05 were considered statistically significant (a priori). Results were further stratified by age groups to highlight age differences and were also broken down by EQ-5D-5L subdomain to determine potential drivers of utility and EQ-VAS differences. To highlight the potential correlations between HRQoL outcomes and population characteristics, ordinary least squares (OLS) linear regressions were performed for utility and VAS measurements. To gain understanding of the impact of the pandemic on population mental health and to identify key drivers affecting HRQoL, ordered/ordinal logistic regressions were conducted by regressing key parameters such as age, fear of COVID-19, gender, education, employment, wave, and country onto the EQ-5D-5L subdomain of anxiety/depression.

We also calculated total quality-adjusted life year (QALY) changes for the US, Swedish, Norwegian populations using the EQ-5D-5L utility scores to evaluate the potential changes in population health. QALY values were calculated by multiplying the average utility from our survey by the total population in each age group according to publicly available census data [18–20], then compared across waves to estimate population level QALYs gained/loss in each country.

## Results

We received a total of 2,734, 1,003 and 1,020 responses in wave 1, and 2,252, 1,013 and 1,011 responses in wave 2 for the US, Sweden, and Norway, respectively (Table 1 and Table 5 in Appendix). Average age was similar in the three countries in both waves, with the mean age being 42.6 years, 47.8 years, 46.9 years in wave 1, and 42.6 years, 46.7 years, 46.9 years in wave 2, for the US, Sweden, and Norway, respectively. Mean utility scores were 0.822, 0.768, and 0.808 in wave 1 (*p* < 0.001), 0.823, 0.783, and 0.777 in wave 2 (*p* < 0.001). Mean rescaled EQ-VAS scores were 0.746, 0.687, and 0.692 in wave 1 (*p* < 0.001), 0.764, 0.682, and 0.678 in wave 2 (*p* < 0.001). For both waves, EQ-5D-5L utilities and EQ-VAS scores in the US remained higher than Sweden and Norway (*p* < 0.001). Between waves, a significant reduction in EQ-5D-5L utility was observed in Norway (0.808 vs. 0.777, *p *< 0.01). On the other hand, a significant increase in EQ-VAS scores was detected in the US (0.746 vs. 0.764, *p* < 0.001). Self-reported problems with anxiety/depression were highest for the US, followed by Sweden. Norwegians reported most problems with pain/discomfort, followed by anxiety/depression.

**Fig. 1:**
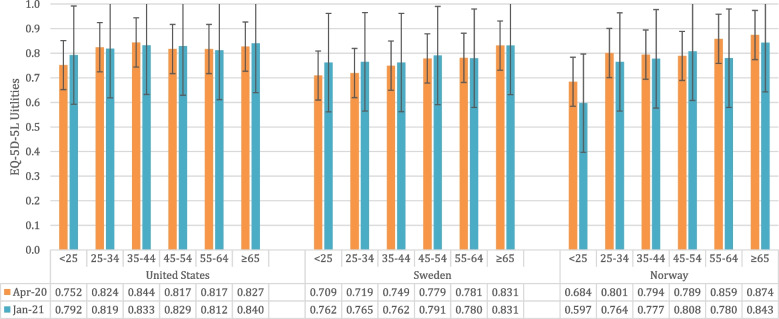
EQ-5D-5L Utility values for both survey waves stratified by country and age group

Norwegians aged 45–54 years was the only age group that showed a slight EQ-5D-5L utility increase in wave 2, while all other age groups showed decreased utilities over time, especially for those younger than 25 years (Fig. 1). On the other hand, although not significant, younger respondents (< 25 years) in the US (*p* = 0.1630) and Sweden (*p* = 0.1931) showed a utility increase over time. Regardless of age group, all mean EQ-VAS scores in the US increased in wave 2, however, this was not the case for Swedish and Norwegians.

**Fig. 2:**
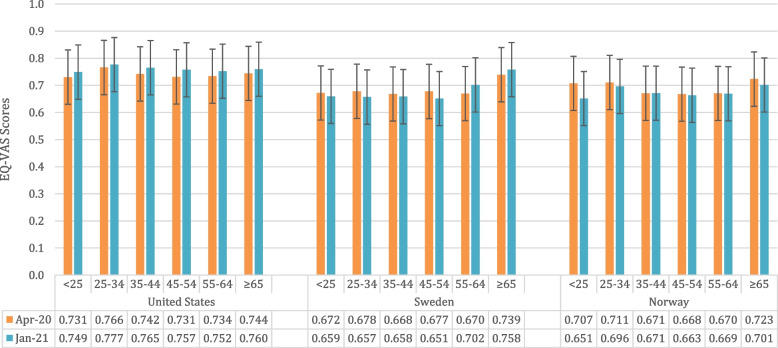
EQ-VAS scores for both survey waves stratified by country and age group

Except for Swedish individuals older than 55 years and Norwegians aged 35–44 years, all Scandinavians reported lower EQ-VAS scores in wave 2. This was particularly the case for the younger age groups, especially for Norwegians younger than 25 years; the reported EQ-VAS scores dropped from 0.707 in wave 1 to 0.651 in wave 2 (Fig. 2, *p* = 0.0732). All US respondents reported higher EQ-VAS scores in wave 2, especially those aged 25 to 34 years and 35 to 44 years (*p* = 0.2516 and *p* = 0.0236).

**Fig. 3:**
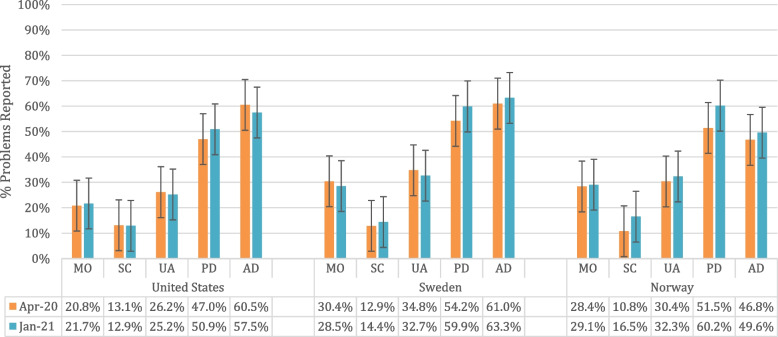
EQ-5D-5L health domain responses for both survey waves stratified by country and age group. Abbreviations: MO, Mobility; SC, Selfcare; UA, Usual Activities; PD, Pain / Discomfort; AD, Anxiety / Depression

The EQ-5D-5L subdomain results are shown in Fig. 3. Among the EQ-5D-5L subdomain responses, anxiety/depression was the most problematic in the US and Sweden for both waves (> 50%), followed by pain/discomfort. Norwegians reported most problems in pain/discomfort followed by anxiety/depression. Proportions of problems in all subdomains increased over time for Norwegians and decreased for Americans. Further, younger respondents had proportionally more problems with anxiety/depression than older respondents, especially for those aged < 25 years. Older respondents, especially for those aged > 65 years always reported less anxiety/depression (< 50%).

We calculated the population QALY changes from April 2020 to January 2021 for all 3 countries (Table 2). Based on pooled estimates, United States and Sweden experienced QALY gains while Norway experienced QALY loss: 1920 for United States, 159 for Sweden and -159 for Norway. Americans younger than 25 and Swedish younger than 35 had the highest QALY gains among all age groups. On the other hand, all age groups in Norway had QALY losses, except for Norwegians aged between 45 and 54.

**Table 2:** Quality-adjusted life year changes of survey participants across survey waves stratified by country and age group

**Country**	**Age Group**	**2020 Population N (thousand)**	**Apr 2020 (Wave 1)**		**Jan 2021 (Wave 2)**			**QALY Difference (thousand)**
			**Mean EQ-5D-5L Utility**	**Estimated Population QALY (thousand)**	**Mean EQ-5D-5L Utility**	**Estimated Population QALY (thousand)**	**Utility Difference**	
**United States**	< 25	42,995^a^	0.752	32,332	0.792	34,052	0.040	1720
	25–34	45,485	0.824	37,480	0.819	37,252	-0.005	-227
	35–44	41,346	0.844	34,896	0.833	34,441	-0.011	-455
	45–54	41,541	0.817	33,939	0.829	34,437	0.012	498
	55–64	42,101	0.817	34,397	0.812	34,186	-0.005	-211
	≥ 65	45,741^b^	0.827	37,828	0.840	38,422	0.013	595
	Pooled QALY Difference							**1920**
**Sweden**	< 25	1,151^a^	0.709	816	0.762	877	0.053	61
	25–34	1,452	0.719	1,044	0.765	1,111	0.046	67
	35–44	1,291	0.749	967	0.762	984	0.013	17
	45–54	1,336	0.779	1,041	0.791	1,057	0.012	16
	55–64	1,214	0.781	948	0.780	947	-0.001	-1
	≥ 65	1,847^b^	0.831	1,535	0.831	1,535	0.000	0
	Pooled QALY Difference							**159**
**Norway**	< 25	659^a^	0.684	451	0.597	393	-0.087	-57
	25–34	745	0.801	597	0.764	569	-0.037	-28
	35–44	704	0.794	559	0.777	547	-0.017	-12
	45–54	748	0.789	590	0.808	604	0.019	14
	55–64	639	0.859	549	0.780	498	-0.079	-50
	≥ 65	825^b^	0.874	721	0.843	695	-0.031	-26
	Pooled QALY Difference							**-159**

*Abbreviations*: *QALY* Quality-adjusted Life Year

^a^Assumed to be population aged between 15 and 24 in the census data;

^b^Assumed to be population aged between 65 and 84 in the census data

Linear regression results of the EQ-5D-5L utility and EQ-VAS scores show that the directions and statistical significance of the coefficients were similar across the three countries (Table 3). Holding other factors constant, for instance, being employed in the US was associated with 0.0308 increase in EQ-5D-5L utility on average. In Sweden, each year increase of age was associated with 0.00209 higher rescaled EQ-VAS score (*p* < 0.001). The degree of fear of COVID-19 negatively impacted both the EQ-5D-5L utilities and EQ-VAS scores for all 3 countries (*p* < 0.001). We also observed significant and negative associations between fear of financial well-being and both HRQoL outcomes in the US and Sweden (*p* < 0.001). Being employed was always associated with better HRQoL outcomes in each country (*p* < 0.001).

**Table 3:** Association between health-related quality of life outcomes and characteristics of survey participants

		**Outcome = EQ-5D-5L Utility (0–1 scale)**				**Outcome = EQ-VAS (0–1 scale)**			
		**United States**	**Sweden**	**Norway**	**Pooled**	**United States**	**Sweden**	**Norway**	**Pooled**
Age		0.000492*	0.00275***	0.00404***	0.00198***	-0.000122	0.00209***	0.000995**	0.000760***
Fear on Health During Pandemic (0–10)		-0.0125***	-0.0134***	-0.0152***	-0.0129***	-0.00747***	-0.00727***	-0.0100***	-0.00758***
Fear on Financial Situation During Pandemic (0–10)		-0.00735***	-0.00823***	-0.00396	-0.00723***	-0.00512***	-0.00738***	-0.00502*	-0.00556***
Gender	Female	(Reference)				(Reference)			
	Male	0.0102	0.0242*	0.00251	0.0155**	0.00705	0.0296**	0.00677	0.0144***
Education	Under College Education	(Reference)				(Reference)			
	College Education	0.0116	0.0188	0.0217	0.0171**	0.0422***	0.0170	0.0413***	0.0365***
Employment	Unemployed	(Reference)				(Reference)			
	Employed	0.0308***	0.0712***	0.117***	0.0629***	0.0503***	0.0626***	0.0857***	0.0627***
Wave	Wave 1 (Apr 2020)	(Reference)				(Reference)			
	Wave 2 (Jan 2021)	0.00133	0.00488	-0.0371**	-0.00701	0.0195***	-0.0134	-0.0198*	0.00228
β0 (Constant)		0.874***	0.682***	0.629***	0.784***	0.749***	0.603***	0.645***	0.713***
Country	United States	N/A	N/A	N/A	(Reference)	N/A	N/A	N/A	(Reference)
	Norway				-0.0491***				-0.0664***
	Sweden				-0.0575***				-0.0612***

^*^ *p* < 0.05, ** *p* < 0.01, *** *p* < 0.001

Tables 4 shows the odds-ratios estimated from the ordered logistic regression on anxiety/depression subdomain. For each unit increase in rating of fear on health during the pandemic, holding other variables in the model constant, the odds of a participant reporting “Slightly Problematic” was > 10% in all countries, especially for Sweden (16.9%), followed by Norway (13%) and the US (10.5%), compared with those who reported “Not Problematic” (*p* < 0.001). Age and employment were significantly correlated with less anxiety/depression problems in all 3 countries. Increase in fear of COVID-19’s impact on financial well-being were significantly associated with worsened anxiety/depression across each country, with 15.2% for the US, 10.9% for Sweden, and 9.9% for Norway. Sweden, on average, was estimated to perform the worst with regard to anxiety/depression subdomain, while the performance of the US and Norway did not differ significantly.

**Table 4:** Association between EQ-5D-5L anxiety/depression subdomain responses and characteristics of survey participants

		Outcome = EQ-5D-5L Anxiety/Depression Subdomain (From No Problems to Extreme Problems)			
		United States	Sweden	Norway	Pooled
Age		0.976***	0.963***	0.965***	0.971***
Fear on health during pandemic (0–10)		1.105***	1.169***	1.130***	1.118***
Fear on financial situation during pandemic (0–10)		1.152***	1.109***	1.099***	1.135***
Gender	Female	(Reference)			
	Male	0.658***	0.718***	0.856	0.698***
Education	Under College Education	(Reference)			
	College Education	0.861*	1.071	0.875	0.910*
Employment	Unemployed	(Reference)			
	Employed	0.742***	0.680***	0.645***	0.705***
Wave	Wave 1 (Apr 2020)	(Reference)			
	Wave 2 (Jan 2021)	0.942	1.092	1.282**	1.047
Country	United States	N/A	N/A	N/A	(Reference)
	Norway				0.930
	Sweden				1.472***
Latent outcome cut points (ancillary parameters)					
Not problematic | Slightly		-0.680	-1.533	-0.989	-0.873
Slightly problematic | Moderately		0.771	0.208	0.487	0.641
Moderately problematic | Severely		2.459	1.431	1.679	2.111
Severely problematic | Extremely		3.451	2.889	3.297	3.334

* *p* < 0.05, ** *p* < 0.01, *** *p* < 0.001

## Discussion

Based on the EQ-VAS, population HRQoL in Sweden and Norway remained similar across waves, while population HRQoL improved in wave 2 for the US, exhibiting a rebound pattern. In contrast, the trends observed based on the EQ-5D-5L utility scores and QALYs showed mixed outcomes.

Self-reported problems associated with anxiety/depressions were by far the most problematic in each country, especially for US and Sweden, followed by pain/discomfort. For Norwegians, anxiety/depression was the second most significant health problem, following pain/discomfort. Self-reported anxiety/depression problems were strongly associated with age as indicated by greater proportions of problems reported by younger age groups. Such results suggest that the mental health impact of the COVID-19 pandemic falls primarily on younger populations. For example, many young people expected to attend college or start new careers in Spring of 2020, and the pandemic disrupted many of these plans. In contrast, it is more likely that older individuals have established routines and careers, suggesting that the pandemic had a less disruptive impact on HRQoL among this age group.

**Fig. 4:**
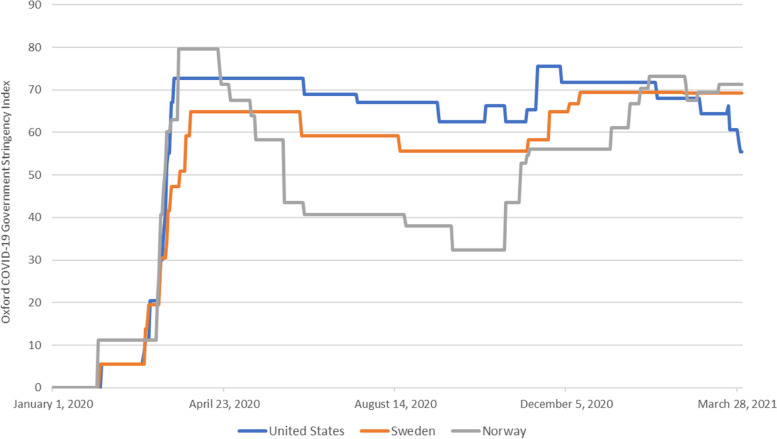
Oxford COVID-19 stringency index between Jan 2020 and April 2021 in the United States, Sweden, and Norway based on Hale et al., (2021). Note: The metrics (Hale et al., 2021) used to calculate the Government Stringency Index include school closures, workplace closures, cancellation of public events, restrictions on public gatherings, closures of public transport, stay-at-home requirements, public information campaigns, restrictions on internal movements, and international travel controls

Differences between each country may be correlated with different pandemic containment policies implemented at each time point of our study. For instance, the Oxford COVID-19 government response stringency index is a metric scaled from 0 to 100 (0 being the least strict and 100 the strictest) calculated based on various government responses during the pandemic, such as school closures and travel bans [9]. According to the index, Norway initially had the strictest policies among the three countries during March 2020, but soon became the country with the least strict policies for about one year until around Feb 2021. In comparison, the United States had stricter policies than both Scandinavian countries initially then became less stringent over time. By March 2021, the US had the least stringent mitigation policies compared with both Sweden and Norway (Fig. 4 in Appendix) [9].

It Is also important to highlight that the EQ-VAS scores represent a respondent’s self-rating of health today, while EQ-5D-5L utility scores represent a weighted rating of HRQoL using a country-specific algorithm to generate the score based on societal preferences on the basis of the defined 5 health subdomains. Hence, the EQ-VAS can measure latent health impairments that a respondent experiences, while the EQ-5D-5L captures latent health as defined by the five subdomains. This may explain the discrepancies in trends between the two waves when comparing the EQ-VAS scores to EQ-5D-5L utility values. For instance, in the United States, the timing of Wave 2 occurred shortly after the inauguration of incoming President Joe Biden and after the lifting of strict measures in certain states, perhaps explaining why the EQ-VAS scores generally showed significant improvements across all age groups, while the EQ-5D-5L utility scores did not show the same magnitude of effect over time. In the Scandinavian countries, there was a tightening of restrictions after implementing fairly lax mitigation policies during the early phases of the pandemic. It is possible that “pandemic fatigue” played a role.

Our regression results showed that employment had a positive effect on all HRQoL measures including EQ-VAS, EQ-5D-5L utilities, as well as the anxiety/depression subdomain in all three countries. Fear of the pandemic’s impact on health and financial situation consistently showed a very negative effect on HRQoL measures in all three countries. These results are consistent with literature findings that employment can improve mental health[21], especially since continued employment is inherently associated with greater financial security, not to mention the employment also suggests one’s physical ability to work, which in turn would have important effect on one’s overall HRQoL, including mental health. These findings suggest that public health policy for future pandemics should consider the importance of continued employment for individuals, in addition to extended unemployment benefits.

We acknowledge that this study is limited by an online sample, relatively short study period and self-reported health measurements. In Sweden and Norway, sample selection was based on broadly nationally representative samples of age and sex, while in the United States, although we attempted to capture a longitudinal cohort between waves, there was still significant rates of attrition [2, 22]. Furthermore, in the United States, the crowdsourcing online data collection platform, the Amazon Mturk, has been shown to have limited external validity to the general population, depending on context and study type [23–27]. Nevertheless, literature evidence also suggested that Amazon Mturk respondents are at least more representative of the US population than in-person convenience samples, and thus may be used for research purpose [28–30]. The approach of treating EQ-VAS results as interval data were also shown to have more limitations than other methods [31]. Nonetheless, our work is significant in capturing direct HRQoL comparisons across the three countries that implemented mitigation policies with varying stringency, resulting in different level of impacts on population HRQoL outcomes.

## Conclusion

Population HRQoL improved after the first year of the pandemic in the United States based on the EQ-VAS, but not in Sweden and Norway. Sustained large proportions reporting problems in anxiety/depression in both waves and in all three countries suggests that population mental health effects of the pandemic are a major concern, especially among the younger age groups. Employment stability was significantly associated with better HRQoL outcomes and may be a public health policy target worth considering in the future.

## Data Availability

The datasets used and/or analyzed during the current study are available from the corresponding author on reasonable request.

## Appendix

**Table 5:** Characteristics of survey participants stratified by country and survey wave

	**United States**			**Sweden**			**Norway**		
	**Wave 1**	**Wave 2**	***p*** **-value**	**Wave 1**	**Wave 2**	***p*** **-value**	**Wave 1**	**Wave 2**	***p*** **-value**
**N**	2,734	2,252	N/A	1,003	1,013	N/A	1,020	1,011	N/A
**Age (Mean, SD)**	42.6 (14.3)	42.6 (13.9)	0.9447	47.8 (17.1)	46.7 (16.6)	0.1541	46.9 (17.0)	46.9 (16.6)	0.9673
**Female (n, %)**	1,361 (49.9)	1,101 (48.9)	0.5152	508 (50.7)	514 (50.7)	0.9669	524 (51.4)	518 (51.2)	0.9511
**College education (n, %)**	1,999 (73.1)	1,447 (64.3)	< 0.001	307 (30.6)	357 (35.2)	0.0269	347 (34.0)	344 (34.0)	0.9977
**Employment (n, %)**	2,018 (74.8)	1,737 (78.2)	0.0056	519 (51.7)	569 (56.2)	0.0463	506 (49.6)	513 (50.7)	0.6093
**Fear on health during pandemic (Mean, SD)**	5.20 (2.95)	5.50 (3.07)	< 0.001	4.65 (2.88)	4.36 (2.64)	0.0206	4.05 (2.77)	3.88 (2.65)	0.1656
**Fear on financial situation during pandemic (Mean, SD)**	5.79 (3.00)	5.36 (3.13)	< 0.001	4.49 (2.89)	3.86 (2.75)	< 0.001	3.82 (2.74)	3.29 (2.69)	< 0.001
**EQ-5D-5L Utilities (Mean, SD)**	0.822 (0.222)	0.823 (0.221)	0.7904	0.768 (0.260)	0.783 (0.237)	0.1785	0.808 (0.248)	0.777 (0.271)	0.0067
**Scaled EQ-VAS (Mean, SD)**	0.746 (0.192)	0.764 (0.186)	< 0.001	0.687 (0.214)	0.682 (0.203)	0.5638	0.692 (0.208)	0.678 (0.217)	< 0.001

## Abbreviations

HRQoL: Health-related quality of life
VAS: Visual analog scale
QALY: Quality-adjusted life year
MO: Mobility
SC: Selfcare
UA: Usual Activities
PD: Pain / Discomfort
AD: Anxiety / Depression
